# Discrepancy between short-term and long-term effects of bone marrow-derived cell therapy in acute myocardial infarction: a systematic review and meta-analysis

**DOI:** 10.1186/s13287-016-0415-z

**Published:** 2016-10-20

**Authors:** Seon Heui Lee, Jin Hyuk Hong, Kyoung Hee Cho, Jin-Won Noh, Hyun-Jai Cho

**Affiliations:** 1 Department of Nursing Science, College of Nursing, Gachon University, Seoul, South Korea; 2 Department of Biostatistics, Korea University College of Medicine, Seoul, South Korea; 3 Department of Public Health, Graduate School, Yonsei University, Seoul, South Korea; 4 Department of Healthcare Management, Eulji University, 212 Yangji-dong, Sujeong-gu, Seongnam-si, Gyeonggi 461-713 South Korea; 5 Cardiovascular Center, Department of Internal Medicine, Seoul National University Hospital, 101 Daehak-ro, Jongno-gu, Seoul 03080 South Korea

**Keywords:** Cell therapy, Acute myocardial infarction, Survival, Bone marrow

## Abstract

**Background:**

Bone marrow-derived cell therapy has been used to treat acute myocardial infarction. However, the therapeutic efficacy of this approach remains controversial. Here, we performed a systematic review and meta-analysis to evaluate short-term and long-term effectiveness of bone marrow-derived therapy.

**Methods:**

We searched eight databases (Ovid-Medline, Ovid-EMBASE, Cochrane Library, KoreaMed, KMBASE, KISS, RISS, and KisTi) up to December 2014. Demographic characteristics, clinical outcomes, and adverse events were analyzed. We identified 5534 potentially relevant studies; 405 were subjected to a full-text review. Forty-three studies with 2635 patients were included in this review.

**Results:**

No safety issues related to cell injection were reported during follow-up. At 6 months, cell-injected patients showed modest improvements in left ventricular ejection fraction (LVEF) compared with the control group. However, there were no differences between groups at other time points. In the cardiac MRI analysis, there were no significant differences in infarct size reduction between groups. Interestingly, mortality tended to be reduced at the 3-year follow-up, and at the 5-year follow-up, cell injection significantly decreased all-cause mortality.

**Conclusions:**

This meta-analysis demonstrated discrepancies between short-term LV functional improvement and long-term all-cause mortality. Future clinical trials should include long-term follow-up outcomes to validate the therapeutic efficacy of cell therapy.

## Background

Despite advances in medical therapy and coronary revascularization treatment, ischemic heart disease remains a major cause of morbidity and mortality worldwide. Investigation of the new therapies to improve cardiac function and clinical outcomes after acute myocardial infarction (AMI) is actively ongoing. Bone marrow (BM)-derived cell therapy has been investigated experimentally in the context of regenerating or repairing the damaged heart and vessels [1, 2], and since 2001 [3–5], more than 100 cell therapy trials, mainly using bone marrow mononuclear cells (BM-MNCs), have been performed in patients with AMI, establishing the safety and clinical feasibility of this cell therapy. However, individual studies are not sufficiently powered to detect any significant differences in major adverse clinical events between the cell therapy and control groups. Therefore, meta-analyses could address the weaknesses of each study and may provide insights into the clinical outcomes and benefits of cell therapy.

Most trials have enrolled small numbers of patients and reported short-term follow-up results, leading to inconclusive and inconsistent results in the detection of significant differences in major adverse clinical events between the cell therapy and control groups. To overcome the limitations of individual studies and increase statistical power, several meta-analyses have been performed. A meta-analysis in 2012 showed that intracoronary BM cell therapy after AMI significantly improved the left ventricular ejection fraction (LVEF) at 6 months after treatment; however, the absolute value of improvement was modest (2.87 %) [6]. In contrast, a meta- analysis in 2014 reported that there was no detectable therapeutic benefit with regard to major adverse cardiac event rates after BM cell therapy after a median follow-up duration of 6 months [7], and a recent meta-analysis in 2015, using individual patient data from 12 randomized trials, demonstrated no benefit for 1-year follow-up LVEF and clinical outcomes [8]. Taken together, these studies suggest that there could be a discrepancy between the improvement of LV function and clinical outcomes. Furthermore, long-term follow-up results are lacking.

Accordingly, we conducted the largest meta-analysis of this topic reported to date, including 43 randomized trials with 2635 patients, and focused on follow-up results at 6 months, 1 year, 3 years, and 5 years in order to evaluate short-term and long-term effects of the cell therapy in patients with AMI. We found the significant difference between cardiac magnetic resonance imaging (MRI) and echocardiographic measurements of LV functional improvement and infarction size reduction. Furthermore, we revealed the discrepancy between the LV function and clinical outcomes. Cell therapy showed the long-term mortality benefits at 5-year follow-up, although the effects of LV functional improvement and infarct size reduction were modest in short-term analyses.

## Methods

### Information sources and search strategy

We searched eight databases (Ovid-Medline, Ovid-EMBASE, Cochrane Library, KoreaMed, KMBASE, KISS, RISS, and KisTi) up to December 2014. To ensure a sensitive search, we designed strategies that included medical subject headings (MeSH) keywords, such as “myocardial infarction”, “acute MI”, “MI”, “STEMI”, “coronary heart disease”, “angina”, “ischemic heart disease”, “ischemic cardiomyopathy”, “heart failure”, “bone marrow cell (BMC)”, “mononuclear cell (MNC)”, “mesenchymal stem cell”, “mesenchymal stromal cell”, “MSC”, “granulocyte colony-stimulating factor (G-CSF)”, and all possible combinations.

### Inclusion and exclusion criteria

**Fig. 1:**
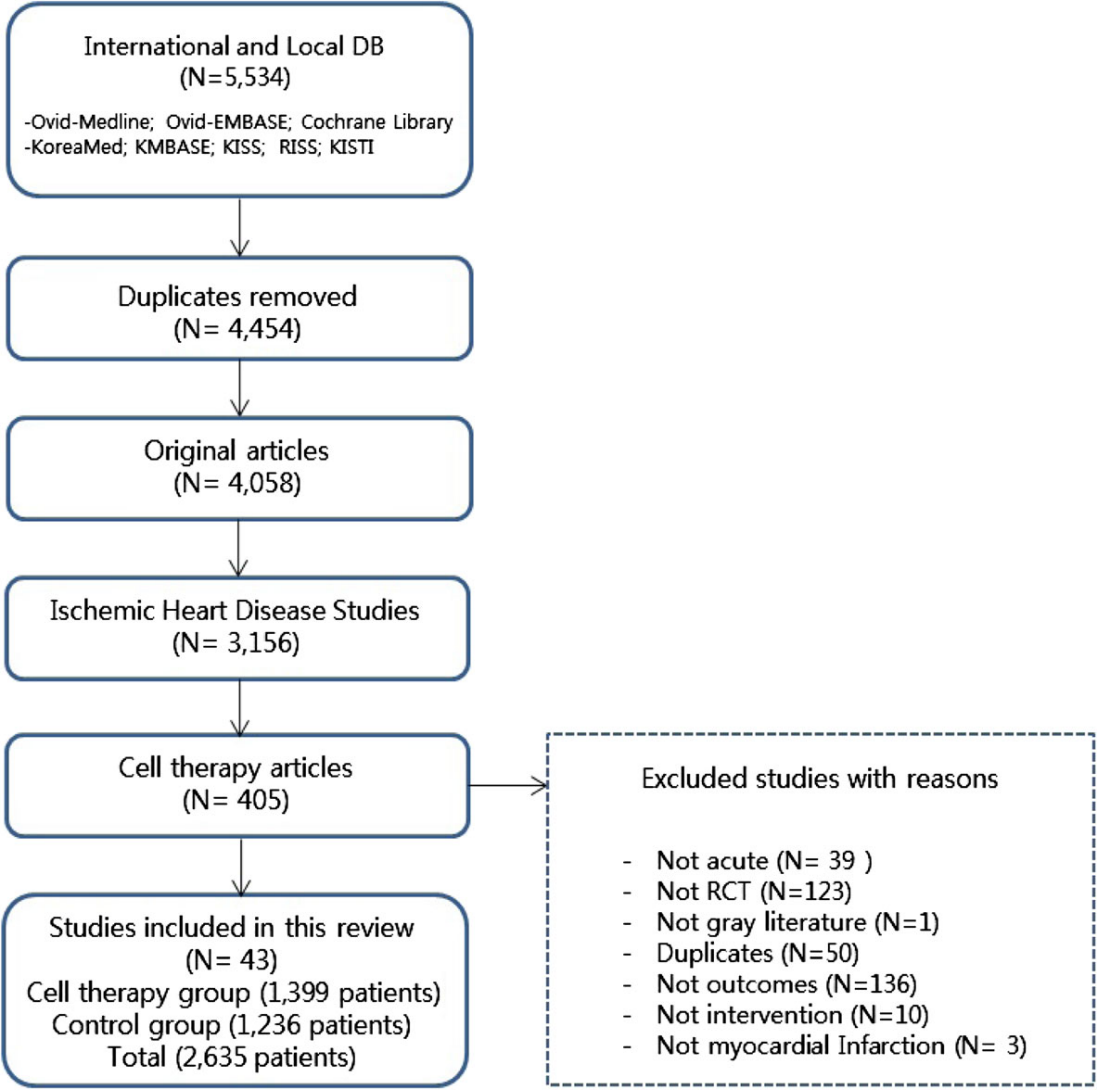
Flow diagram of the literature selection process and meta-analysis

The inclusion criteria were as follows: (1) randomized clinical trials (RCTs), (2) published original articles, (3) written in English, (4) stem cell or cell therapy studies, and (5) included proper outcomes (follow-up LV function, mortality, etc.). The exclusion criteria were as follows: (1) nonhuman studies, (2) preclinical studies, (3) gray literature, (4) non-RCTs, and (5) duplicated reports. Two reviewers screened studies according to the selection criteria. First, we removed duplicate studies and performed title and abstract screening. Second, we selected potentially pertinent studies and reviewed the full text. Finally, we chose 43 randomized clinical trials and analyzed 2635 patients [9–51] (Fig. 1).

### Data extraction and quality assessment

Two independent reviewers assessed studies using a standardized form. Information extracted from the chosen studies included authors, publication year, country, design, results, and funding. Clinical outcomes consisted of complications such as arrhythmia, heart failure, ischemic heart disease (IHD) recurrence, restenosis, revascularization, stroke, and all-cause mortality. Effectiveness outcomes were improvement of left ventricular ejection fraction (LVEF) and reduction of infarct size at each follow-up point. Assessment of the quality of articles was made according to the Cochrane Handbook [52]. Two authors (Lee and Hong) evaluated all of the studies including methodological quality according to the risk of bias for randomized controlled trials.

### Statistical analysis

We conducted the meta-analysis using Review Manager version 5.3 (Cochrane Collaboration). For dichotomous and continuous variables, the odds ratios (ORs) and weighted mean differences were calculated for groups using a random effects model and inverse variance weighting. Inverse variance weighting is a method of aggregating two or more random variables to minimize the variance of the weighted average [53]. For studies that did not report the actual change as the mean, the standard deviation was calculated using a standardized formula that was previously validated when the baseline and follow-up standard deviation were known. In this study, we reported results according to a random effects model considering heterogeneity, and the publication bias was tested using a Funnel plot [53, 54]. The Chi-square test with significance set at *P* < 0.10 was used to assess statistical heterogeneity of selected studies, and I^2^ statistics were provided to quantify the heterogeneity.

## Results

### Search and selection of BM-derived cell therapy clinical trials

We identified 5534 potentially relevant studies and further screened these studies for eligibility. We selected 405 pertinent cell therapy studies for a full-text review. The outcomes of interest included demographic characteristics, clinical outcomes, and effectiveness outcomes at each follow-up point. Of these 405 studies, 362 were excluded because they were not describing acute MI (*n* = 39), were not RCTs (*n* = 122), were gray literature (*n* = 1), were duplicate reports (*n* = 50), did not include clinical outcomes (*n* = 136), were not interventional studies (*n* = 10), and were not MI studies (*n* = 3). We finally included 43 studies with 2635 patients [9–51] (Fig. 1).

### Study characteristics

The studies were published from 2004 to 2014. Table 1 lists the characteristics all of the included studies. The study sizes ranged from 8 to 204 patients, and the follow-up duration ranged from 3 to 108 months. Twenty-four of 41 studies were conducted in Europe, six studies were conducted in the USA, five studies were conducted in China, three studies were conducted in Korea, one study was conducted in Mexico, one study was conducted in Canada, one study was conducted in Thailand, one study was conducted in Brazil, and one study was conducted in Iran. Among the 2635 patients, 1399 patients received BM-derived cell therapy, and 1236 patients were in the control group. Most studies used freshly isolated BM-MNCs isolated by density gradient separation of autologous BM aspirates. Three studies used BM-derived CD133+ cells by cell sorting using a specific antibody [20, 28, 30], and one study used CD34+ cells [24]. Two studies used granulocyte colony-stimulating factor (G-CSF)-mobilized peripheral blood mononuclear cells (PB-MNCs) by leukapheresis [17, 50], and two other studies used mesenchymal stem cells (MSCs), which were cultured from BM aspirates under attached conditions for 1 month [11, 35]. Most cell injections were performed through intracoronary infusion 2 to 7 days after percutaneous coronary intervention (PCI). One study infused cells intravenously after PCI [35] and another study injected cells intramuscularly through the epicardium during coronary artery bypass graft operation [28]. The total number of injected cells is listed in Table 1.

**Table 1:** Study characteristics

Author	Year	Country	Timing of cell injection	Cell injection (*n*)	Control (*n*)	Total patient (*n*)	Cell type	Cell delivery route	Cell preparation	No. of injected cells	Follow-up (months)
Assmus et al. [9]	2014	Germany	After PCI	101	103	204	MNC	IC	Ficoll gradient	1 × 10^6^	60
Benedek et al. [10]	2014	Romania	After PCI	9	9	18	MNC	IC	Concentration by apheresis	1.66 ± 0.32 × 10^9^	48
Lee et al. [11]	2014	Korea	After PCI	30	28	58	MSC	IC	1-month culture	7.2 ± 0.90 × 10^7^	6
Robbers et al. [12]	2014	Netherlands	After PCI	30	45	75	BM-MNC/PB-MNC	IC	Lymphoprep™	296 ± 164 × 10^6^	4
Gao et al. [13]	2013	China	After PCI	21	22	43	MSC	IC	2-week culture	3.08 ± 0.52 x 10^6^	6, 12, 24
Surder et al. [14]	2013	Switzerland	After PCI	65	67	132	MNC	IC	Density gradient	5 × 10^7^ - 5 × 10^8^	4
Wohrle et al. [15]	2013	Germany	After PCI	29	13	42	MNC	IC	Ficoll gradient	324 × 10^6^	6, 12, 24, 36
Jazi et al. [16]	2012	Iran	After PCI	16	16	32	MNC	IC	Ficoll gradient	24.6 ± 8.4 × 10^8^	6
Kang et al. [17]	2012	Korea	After PCI	57	60	117	PB-MNC	IC	G-CSF/leukapheresis	1.1 ± 0.5 × 10^9^	6, 24, 60
Skalicka et al. [18]	2012	Czech	After PCI	17	10	27	MNC	IC	Gelfusine	26.4 × 10^8^	4, 24
Traverse et al. [19]	2012	USA	After PCI	79	41	120	MNC	IC	Sepax®	150 × 10^6^	6
Colombo et al. [20]	2011	Italy	After PCI	5	5	10	CD133+ MNC	IC	CD133 by CliniMACS®	4.9-135 × 10^6^	12
Hirsch et al. [21]	2011	Netherlands	After PCI	69	65	134	MNC	IC	Lymphoprep™	296 ± 164 × 10^6^	4
Pena-Duque et al. [22]	2011	Mexico	After PCI	4	4	8	MNC	IC	Sepax®	1 ~ 2 × 10^6^ CD34+	6
Plewka et al. [23]	2011	Poland	After PCI	40	20	60	MNC	IC	Safe Flow®	1.44 ± 0.49 × 10^8^	24
Quyyumi et al. [24]	2011	USA	After PCI	16	15	31	CD34+ MNC	IC	Isolex 300i®	5 × 10^6^	6, 12
Roncalli et al. [25]	2011	France	After PCI	52	49	101	MNC	IC	Ficoll	98 ± 8.7 × 10^6^	3
Srimahachota et al. [26]	2011	Thailand	After PCI	11	12	23	MNC	IC	Isoprep®	420 ± 221 × 10^6^	6
Turan et al. [27]	2011	Germany	After PCI	38	18	56	MNC	IC	Point of Care system	1 × 10^6^	3, 12
Yerebakan et al. [28]	2011	Germany	During CABG	20	20	40	CD133+ MNC	IM	CD133 by CliniMACS®	6.0 × 10^6^	108
Grajek et al. [29]	2010	Poland	After PCI	31	14	45	MNC	IC	Ficoll gradient	4.1 ± 1.8 × 10^8^	3, 6, 12
Mansour et al. [30]	2010	Canada	After PCI	14		14	CD133+ MNC	IC	CliniMACS®	10 × 10^6^-	4
Piepoli et al. [31]	2010	Italy	After PCI	19	19	38	MNC	IC	Ficoll gradient	418 × 10^6^	3, 6, 12
Traverse et al. [32]	2010	USA	After PCI	30	10	40	MNC	IC	Ficoll gradient	100 × 10^6^	6
Wohrle et al. [33]	2010	Germany	After PCI	29	13	42	MNC	IC	Ficoll gradient	381 ± 130 × 10^6^	6
Cao et al. [34]	2009	USA	After PCI	41	45	86	MNC	IC	Lymphoprep™	5 ± 1.2 × 10^7^	48
Hare et al. [35]	2009	USA	After PCI	39	21	60	MSC (allogeneic)	IV	1-month culture	0.5 ~ 5 × 10^6^	6
Nogueira et al. [36]	2009	Brazil	After PCI	14	6	20	MNC	IC	Ficoll gradient	10 × 10^6^	6
Yao et al. [37]	2009	China	After PCI	12	12	24	MNC	IC	Ficoll gradient	1.9 ± 1.3 × 10^8^	6, 12
Huikuri et al. [38]	2008	Finland	After PCI	40	40	80	MNC	IC	Ficoll gradient	4.02 ± 1.96 × 10^6^	6
Meluzin et al. [39]	2008	Czech	After PCI	20	20	40	MNC	IC	Histopaque1077	1-10 × 10^7^	3, 6, 12
Panovsky et al. [40]	2008	Czech	After PCI	13	17	30	MNC	IC	Histopaque1077	1-10 × 10^8^	3
de Lezo et al. [41]	2007	Spain	After PCI	10	10	20	MNC	IC	Ficoll gradient	9 ± 3 × 10^8^	3
Ge et al. [42]	2006	China	After PCI	10	10	20	MNC	IC	Ficoll gradient	4 × 10^7^	6
Janssens et al. [43]	2006	Belgium	After PCI	33	34	67	MNC	IC	Ficoll gradient	172 ± 72 × 10^6^	4
Lunde et al. [44]	2006	Norway	After PCI	47	50	97	MNC	IC	Ficoll gradient	68 × 10^6^	6, 36
Meluzin et al. [45]	2006	Czech	After PCI	22	22	44	MNC	IC	Histopaque1077	1x10^7^ -1 × 10^8^	3
Schachinger et al. [46]	2006	Germany	After PCI	101	103	204	MNC	IC	Ficoll gradient	236 ± 174 × 10^6^	24
Karpov et al. [47]	2005	Russia	After PCI	22	22	44	MNC	IC	Histopaque1077	88.5 ± 49.2 × 10^6^	6
Ruan et al. [48]	2005	China	After PCI	9	11	20	MNC	IC	Not described	Not described	6
Chen et al. [49]	2004	China	After PCI	34	35	69	MSC	IC	10-day culture	2-5 × 10^6^	3, 6
Kang et al. [50]	2004	Korea	After PCI	6	7	13	PB-MNC	IC	G-CSF/leukapheresis	1 × 10^9^	6, 24
Wollert et al. [51]	2004	Germany	After PCI	30	30	60	MNC	IC	4 % gelatine-polysuccinate	24.6 ± 9.4 × 10^8^	6, 18, 60

Included studies were all randomized clinical trials (RCTs). Plus-minus value indicates mean ± SE

*PCI* percutaneous coronary intervention, *MNC* mononuclear cell, *IC* intracoronary cell infusion, *BM-MNC* bone marrow-derived mononuclear cell, *PB-MNC* peripheral blood-derived mononuclear cell, *G-CSF* granulocyte colony-stimulating factor, *CABG* coronary artery bypass graft, *IM* intramyocardial cell injection, *IV* intravenous cell infusion, *MSC* mesenchymal stromal cell

### Functional improvement of the LV and reduction of infarct size

**Fig. 2:**
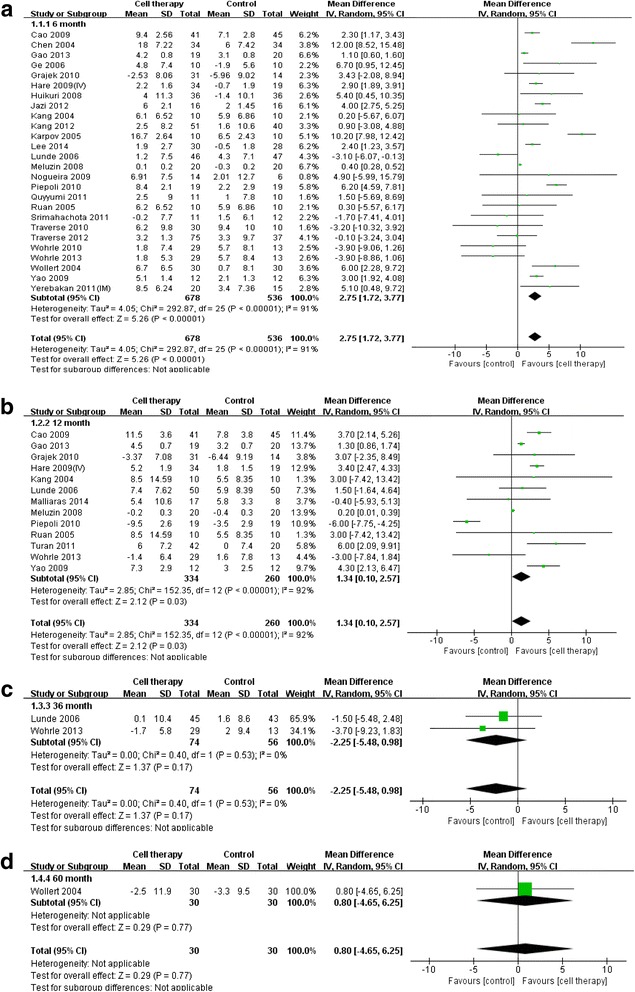
Improvement of left ventricular function. Forest plot and meta-analysis of the left ventricular ejection fraction (LVEF) at the 6-month, 1-year, 3-year, and 5-year follow-up. The weighted mean differences were calculated for groups using a random effects model and inverse variance weighting. Inverse variance weighting is a method for aggregating two or more random variables to minimize the variance of the weighted average. **a** LVEF at 6 months after treatment. **b** LVEF at 1 year. **c** LVEF at 3 years. **d** LVEF at 5 years

**Fig. 3:**
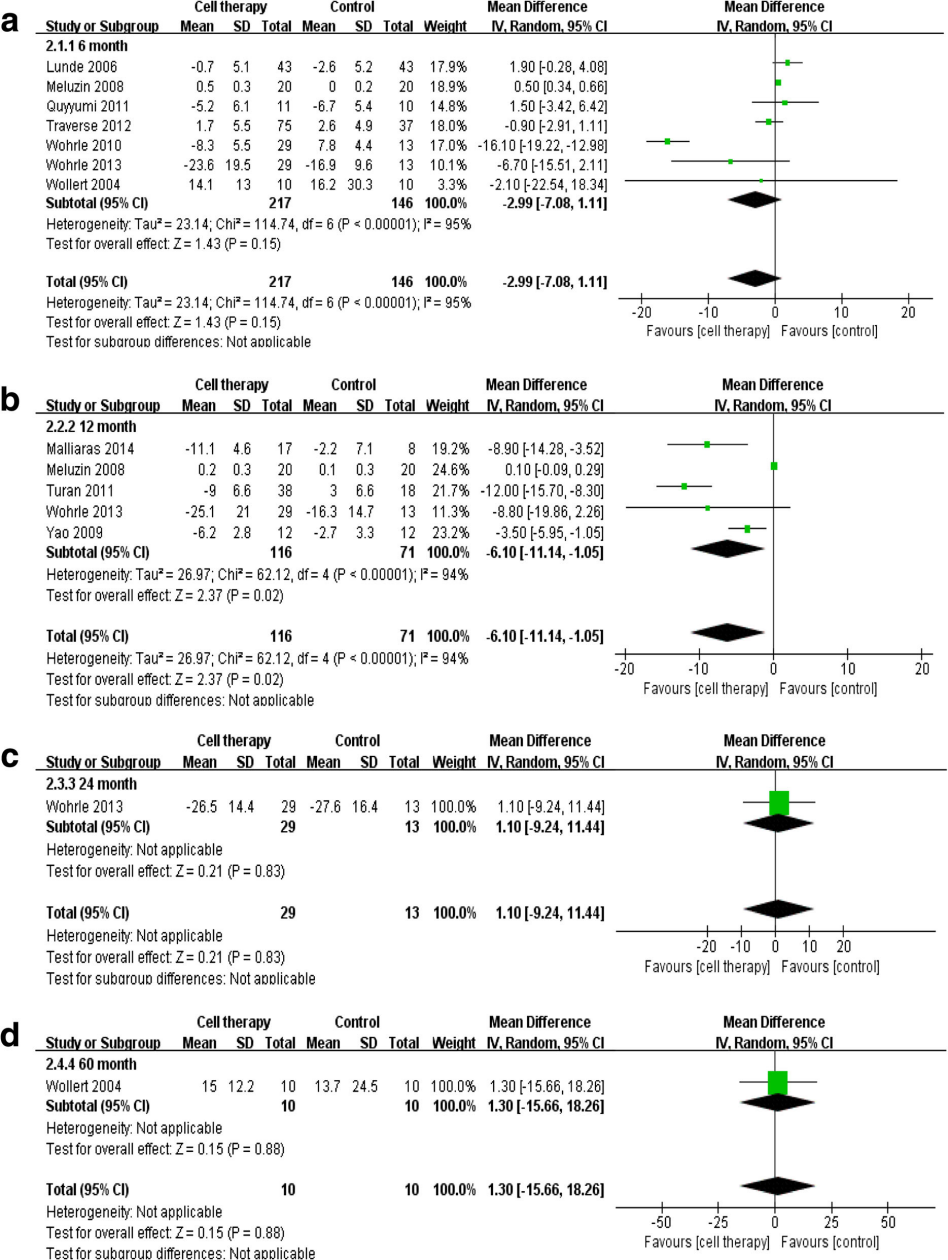
Infarct size reduction. Forest plot and meta-analysis of infarct size at the 6-month, 1-year, 3-year, and 5-year follow-up. **a** Infarct size at 6 months after treatment. **b** Infarct size at 1 year. **c** Infarct size at 3 years. **d** Infarct size at 5 years

Analyses based on a random effects model for the difference in LVEF and infarct size are shown in Fig. 2. Cell therapy improved the LVEF at 6 months (2.75 % increase; *P* < 0.001) and 1 year (1.34 % increase; *P* = 0.03) as compared with the control group (Fig. 2a and b). However, at the 3- and 5-year follow-up, there were no significant differences in LVEF between the cell therapy and control groups (Fig. 2c and d). Infarct size in the cell therapy tended to decrease at 6 months compared with that in the control group; however, this difference was not statistically significant (−2.99 %; 95 % confidence interval [CI],−7.08, 1.11; *P* = 0.15; Fig. 3a). At the 1-year follow-up, infarct size was significantly reduced (−6.10 %; 95 % CI, −11.14, −1.05; *P* = 0.02; Fig. 3b). However, there were no significant differences in infarct size at the 3-year (1.10 %; 95 % CI, −9.24, 11.44; *P* = 0.83; Fig. 3c) and 5-year follow-up (1.10 %; 95 % CI, −9.24, 11.44; *P* = 0.83; Fig. 3d).

Taken together, these data indicated that the differences in LV systolic function and infarct size between the cell therapy and standard care control groups disappeared within 1 year of the initial treatment.

### Difference between measurement modalities

**Fig. 4:**
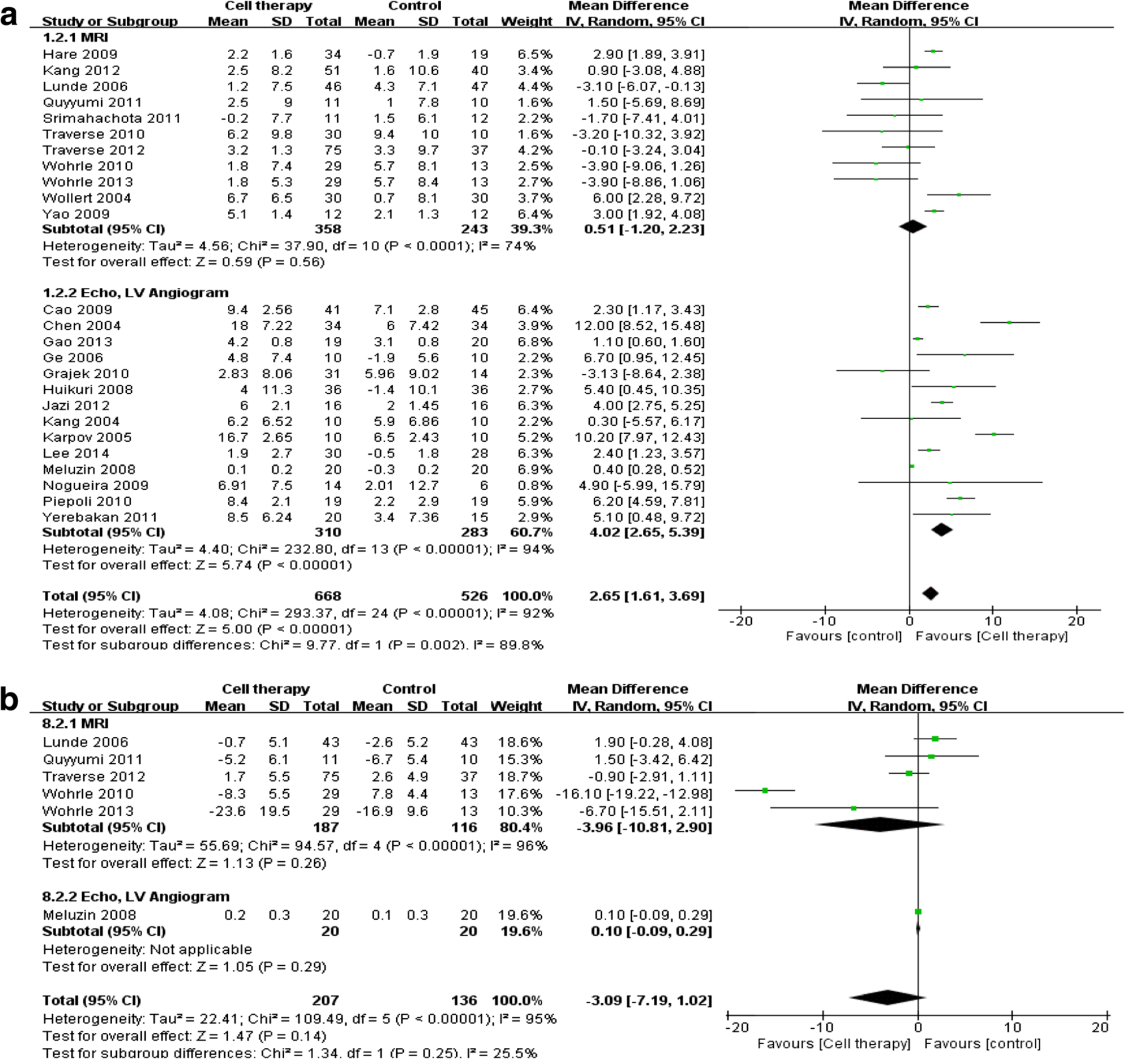
Left ventricular function and infarct size measured by cardiac MRI versus echocardiography or LV angiogram (non-MRI). **a** LVEF at the end point. **b** Infarct size at the end point

It is possible for there to be differences in measurements between the modalities [12]. Accordingly, we performed subgroup analysis of the results from cardiac MRI and echocardiography (or LV angiogram). LVEF measured by cardiac MRI showed no significant difference between the cell therapy and control groups (0.51 %; 95 % CI, −1.20, 2.23; *P* = 0.56), whereas echocardiographic (or LV angiographic) measurement (non-MRI) revealed that cell therapy significantly increased LVEF compared with that in the control group (4.02 %; 95 % CI, 2.65, 5.39; *P* < 0.001; Table 2 and Fig. 4a).

**Table 2:** Subgroup analysis of left ventricular ejection fraction and infarct size at 6 months

Outcome	Subgroup		Studies	Cell-injected group	Control group	Mean difference		I^2^ (%)
				participants (*n*)	participants (*n*)	IV, random (%)	95 % CI	
LVEF	Group 1	MRI	11	358	243	0.51	−1.20, 2.23	74
		Echo or LV angiogram	14	310	283	4.02	2.65, 5.39	94
		Total	25	668	526	2.65	1.61, 3.69	92
	Group 2	Placebo procedure	9	296	195	1.86	0.54, 3.19	64
		No procedure	15	358	325	3.20	1.83, 4.56	94
		Total	24	654	520	2.63	1.59, 3.67	92
Infarct size	Group 1	MRI	5	187	116	−3.96	−10.81, 2.90	96
		Echo or LV angiogram	1	20	20	0.10	−0.09, 0.29	NA
		Total	6	207	136	−3.09	−7.19, 1.02	95
	Group 2	Placebo procedure	3	133	63	−7.93	−19.57, 3.72	97
		No procedure	3	74	73	0.55	−0.62, 1.72	31
		Total	6	207	136	−3.09	−7.19, 1.02	95

*IV* inverse variance, *CI* confidence interval, *I* ^*2*^ inconsistency (across studies), *MRI* magnetic resonance imaging, *LVEF* left ventricular ejection fraction

To examine the effects of study design on maintaining blindness, we also analyzed bone marrow aspiration and placebo infusion in patients in the control group. Patients were separated into two groups according to whether each procedure was used in the control group; cells were isolated from the BM by aspiration and infused placebo in the control group (placebo procedure), or no procedure was performed in the control group (Table 2). The outcome was measured as the change in LVEF from baseline to 6 months. Significant effects were observed the group in which the control group underwent aspiration and placebo infusion, as indicated by an LVEF of 1.86 % (95 % CI, 0.54, 3.19; *P* = 0.006). Additionally, the other group in which no procedures were performed showed significant differences in LVEF (3.20 %; 95 % CI, 1.84, 4.56; *P* < 0.001).

Regarding the reduction in infarct size (Table 2 and Fig. 4b), MRI measurement did not show any significant differences in infarct size (−3.96 %; 95 % CI, −10.81, 2.90; *P* = 0.26) between the cell therapy and control groups. Moreover, non-MRI modalities (echocardiography or LV angiogram) also showed no significant differences (0.1 %; 95 % CI, −0.09, 0.29; *P* = 0.29). No significant treatment effect was found the group in which the control group underwent aspiration in infarct size (−7.93 %; 95 % CI, −19.57, 3.72; *P* = 0.18). Additionally, lack of aspiration in the control group did not have any beneficial effects on infarct size reduction (0.55 %; 95 % CI, −0.62, 1.72; *P* = 0.36).

### Major adverse clinical events

The safety of BM cell harvesting and delivery has been established. All-cause mortality was analyzed by the Mantel-Haenszel (M-H) random model, which is one of the most popular procedures for detecting differential item functioning [55]. If we assume a constant relative Odds (θ_*i*_ = μ or Δ^2^ = 0), then the Mantel-Haenszel statistic is optimal for testing H_0_ : *μ* = 1, and there is considerable literature on efficient estimates of *μ* and on methods for testing H_0_ : *θ*_1_ = *θ*_2_ = … = *θ*_*k*_ [56].

**Fig. 5:**
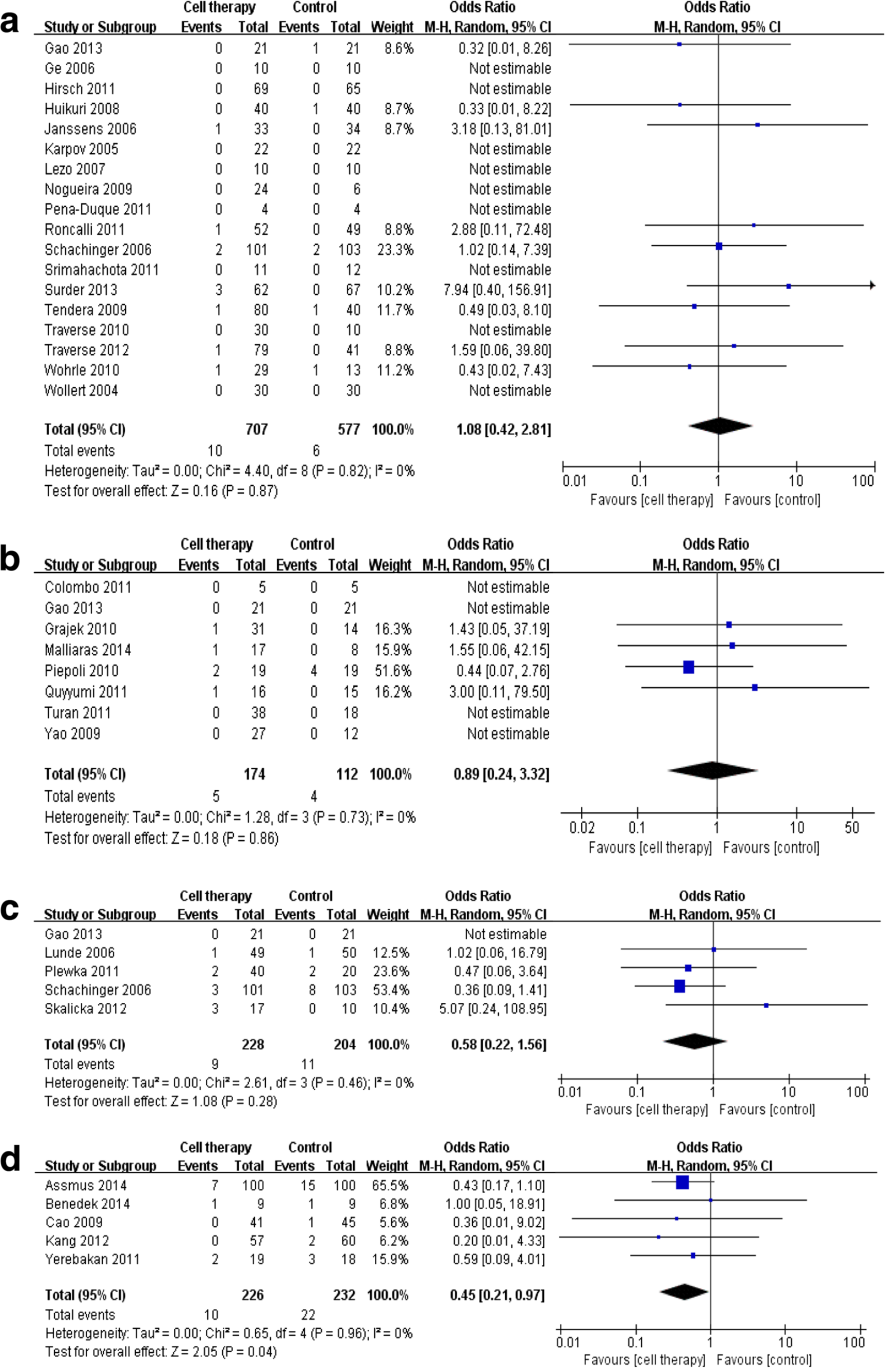
Forest plot and meta-analysis of all-cause mortality. **a** All-cause mortality at 6 months after treatment. **b** All-cause mortality at the 1-year follow-up. **c** All-cause mortality at the 3-year follow-up. **d** All-cause mortality at the 5-year follow-up

The results demonstrated that there were no differences between groups at the 6-month (odds ratio [OR], 1.08; 95 % CI, 0.42, 2.8; *P* = 0.87) and 1-year follow-up (OR, 0.89; 95 % CI, 0.24, 3.32; *P* = 0.86; Table 3 and Fig. 5). Interestingly, mortality tended to decrease at the 3-year follow-up in the cell therapy group compared with that in the control group (OR, 0.58; 95 % CI, 0.22, 1.56; *P* = 0.28). At 5 years, 458 patients were followed up from five studies. BM-derived cell therapy significantly decreased all-cause mortality, with a 55 % relative risk reduction (OR, 0.45; 95 % CI, 0.21, 0.97; *P* = 0.04). All-cause mortality occurred in 4.4 % (10 deaths in 226 patients) in the cell therapy group and 9.5 % (22 deaths in 232 patients) in the control group.

**Table 3:** Major adverse events

Event			Cell therapy group		Control group		Odds ratio	
	Follow-up	Study (participants)	Total event	Participants	Total event	Participants	(M-H, random)	95 % CI
All-cause mortality	6 mo	18 (1284)	10	707	6	577	1.08	0.42–2.81
	1 yr	8 (286)	5	174	4	112	0.89	0.24–3.32
	3 yr	5 (432)	9	228	11	204	0.58	0/22–1.56
	5 yr	5 (458)	10	226	22	232	0.45	0.21–0.97
Heart failure admission	6 mo	9 (970)	10	512	15	458	0.56	0.24–1.31
	1 yr	3 (83)	2	42	0	41	3.07	0.30–30.96
	2 yr	4 (333)	4	179	14	154	0.15	0.04–0.50
	3 yr	6 (577)	11	284	17	293	0.65	0.30–1.44
Recurrence of ischemic heart disease	6 mo	14 (1214)	29	663	37	551	0.57	0.32–1.00
	1 yr	2 (81)	0	48	1	33	0.14	0.01–3.68
	2 yr	6 (373)	6	199	11	174	0.68	0.18–2.56
	3 yr	6 (528)	9	262	11	266	0.81	0.33–2.00
Revascularization/restenosis	6 mo	14 (1212)	98	662	92	550	0.87	0.63–1.22
	1 yr	4 (156)	2	87	4	69	0.37	0.07–1.89
	2 yr	6 (373)	36	199	45	174	0.65	0.39–1.10
	3 yr	6 (577)	62	284	76	293	0.77	0.52–1.15
Cerebral vascular accident (CVA)	1 yr	2 (150)	1	68	2	82	0.69	0.08–5.81
	2 yr	3 (500)	5	248	10	252	0.5	0.16–1.50

*M-H* Mantel-Haenszel, *CI* confidence interval, *mo* months, *yr* years

In the morbidity analysis, including heart failure, recurrence of IHD, repeated revascularization, and stroke (Table 3), the cell therapy group generally showed favorable outcomes; however, this difference was not statistically significant compared with the control.

Taken together, these data indicated that BM-derived cell therapy for patients with AMI produce clinical benefits in the long-term follow-up.

## Discussion

Here, we conducted a systematic review and meta-analysis of comparative studies to evaluate effectiveness of BM-derived cell therapy for patients with AMI. Our study included the greatest number of publications describing cell therapy trials to date and reflects the latest clinical outcomes and long-term follow-up results. We included all types of BM-derived cell therapy trials and analyzed 2635 patients. Among 43 studies, the majority of trials used uncultured fresh autologous BM aspirates and applied a density gradient to separate MNCs. Two trials employed G-CSF-mobilized peripheral blood MNCs, and four trials utilized CD133+ or CD34+ BM-MNCs separated by antibody-based cell sorting. Two trials cultured BM aspirates for 1 month to obtain MSCs. In terms of cell delivery, most studied applied intracoronary infusion through the balloon catheter.

Although the feasibility of cell therapy has been demonstrated in clinical trials, safety should be considered as a priority in future clinical studies. Malignant tumor formation after transplantation of BM MSCs into animal hearts suggests that the unstable chromosomal status of cultured cells could induce serious adverse events [57]. Furthermore, the calcifying activity of uncultured BM cells has also been reported in preclinical animal studies [58]. These data suggest that careful monitoring of cells before transplantation and long-term follow-up for safety should be performed in clinical trials.

The individual studies were not sufficiently powered to detect significant differences in major adverse clinical events between the cell therapy and control group. Therefore, meta-analyses can be used to evaluate appropriate results of each study and may provide insights into clinical outcomes and benefits of cell therapy. In this meta-analysis, we focused on follow-up results at 6 months, 1 year, 3 years, and 5 years in order to evaluate short-term and long-term effects of the cell therapy. We found that at 6 months, the cell-injected group showed a modest or insignificant improvement in LVEF, depending on the measurement modality. However, there was a tendency toward decreased mortality at 3 years. At the 5-year follow-up, cell injection significantly decreased all-cause mortality (55 % relative risk reduction) as compared with that in the control group, indicating a discrepancy between the short-term LV functional improvement and long-term all-cause mortality.

We further reviewed the specific mode of death of all-cause mortality. The REPAIR-AMI study at the 5-year follow-up demonstrated that BM-derived cell therapy tended to reduce cardiac death (4 deaths/100 patients) compared with that of the placebo treatment (14 deaths/100 patients) [9]. Additionally, the MAGIC-Cell study at the 5-year follow-up showed that G-CSF-mobilized cell therapy decreased cardiac death (no deaths/57 patients) compared with that of the control treatment (2 deaths/60 patients) [17]. These data suggested that the effects of cell therapy on mortality could be primarily driven by the reduction of cardiac death after MI.

Functional improvement of LV systolic function was modest, as compared to the standard care of revascularization and medical treatment of MI, BM-derived cell therapy showed better clinical outcomes and survival benefits in the long-term follow-up analysis. We would like to suggest several putative explanations for the significant clinical benefit after cell therapy. First, LV functional improvement and infarct size reduction at the early phase after revascularization with cell therapy could affect long-term outcomes according to the “legacy effect”, which was described in a prior diabetes trial; the difference in glycosylated hemoglobin (HbA1c) levels between intensively and conventionally treated patients disappeared within 1 year of the completion of the trial. Nevertheless, outcomes continued to favor the intensively treated group [59]. This meta-analysis suggests that LVEF at short-term follow-up after cell therapy could affect long-term cardiovascular outcomes. REPAIR-AMI long-term follow-up at 2 and 5 years demonstrated favorable clinical outcomes, including cardiovascular death and rehospitalization for heart failure. Specifically, lower LVEF and increased LV end systolic volume at 4 months were associated with adverse long-term outcomes [9, 60]. These data indicated that the functional improvement of LV at the early stage after cell transplantation would induce long-term benefits, although there was no significant difference in LVEF at long-term follow-up. Furthermore, to overcome such limitations of a single cell transplantation procedure and to achieve better outcomes, repeated cell transplantation is currently under investigation [61]. Second, there is a possibility of sustained LV functional improvement with a small degree after cell therapy. Although this meta-analysis showed no significant differences in LV function and infarct size at the 3- and 5-year follow-up between the cell therapy and control groups, a previous report suggested that the improvement in LVEF may be maintained over a long-term follow-up of 1–5 years [6]. Third, microvascular improvement thorough enhanced angiogenesis and cardioprotective effects could be contributed to outcomes. However, an MRI study reported that cell therapy did not augment the perfusion of the ischemic myocardium [12]. Fourth, patients with recovered LV function after standard revascularization therapy, considered as a low-risk population, could dilute benefits of cell therapy. In the REPAIR-AMI trial, patients with an LVEF below 48 % after the primary PCI showed LV functional improvement after cell infusion [62]. There was no difference between the cell therapy and control group in patients with LVEF above 48 %. In addition, CD133+ BM-derived cell therapy during coronary artery bypass grafting (CABG) demonstrated that patients with a pre-operative LVEF of less than 40 % gained more LV functional improvement after cell therapy as compared with that in patients with LVEF higher than 40 % [28]. Taken together, these data suggested that patients with greater damage from AMI could receive greater benefits from BM-derived cell therapy.

More than a decade after the initial cell therapy trial for MI, even though considerable knowledge has been accumulated, there are still many challenges to achieving favorable clinical outcomes [63]. The inconsistent clinical results of autologous BM cells may be due to the considerable patient-to-patient variability related to a decrease in the number and potency of stem and progenitor cells. These discrepancies may also be caused by the limitations of functional LV measurement. Although cardiac MRI is considered the gold standard for measurement of the LVEF, volumes and infarct size, it is difficult to distinguish the infarct from myocardia edema using MRI. Moreover, the cell processing strategy is also a controversial factor that may yield inconsistent clinical results.

The mechanisms mediating the benefits of cell injection remain unclear. In molecular and cellular analyses of BM-derived cell therapy, recent accumulating data have revealed the limited therapeutic efficacy of this strategy. Although BM cells have shown cardiovascular differentiation after transplantation into infarcted hearts, transdifferentiation is considered a rare event [64]. In vivo cell tracking studies in patients using FDG-PET have demonstrated that less than 1 % of delivered cells stay in the myocardium after 24 hours of cell injection, indicating poor engraftment [65]. Therefore, paracrine effects of injected BM cells via the release of several humoral factors have also been proposed as the main mechanism of action [66]. However, autologous cells from diseased and elderly patients impair cellular function for regeneration and repair of infarcted hearts. To overcome such limitations, clinical trials investigating resident cardiac progenitor cell-based therapy and allogeneic healthy donor-derived cell therapy as well as repeated cell administration are ongoing to determine the merits of these second-generation cell therapies.

### Limitations

This study has several limitations. First, pooled data for the meta-analysis showed different baseline characteristics between the cell therapy and placebo groups, which likely reduces the comparability of the meta-analysis. Furthermore, according to the specific study protocol of each study, there were several differences in procedures for cell harvesting, separation, mode of delivery, and timing of delivery. Second, the number of clinical events was small during the follow-up period, although patients suffered from AMI. In 5 years after enrollment, a total of 32 deaths occurred in 458 patients (4.4 % mortality rate in the cell therapy group and 9.5 % mortality rate in the control group). This low event rate could be explained by the exclusion of high-risk patients in most trials, such as those with severe LV dysfunction and heart failure after large myocardial infarction. Clinical events in heart failure patients who were hospitalized due to decompensation have been reported to be approximately 6 % for in-hospital deaths, and even in patients who were discharged alive, the 6-month all-cause mortality rate was 10 % [67]. Accordingly, cell therapy trials for high-risk patients with AMI should be performed to clearly address the clinical benefits of the new therapeutic modality.

## Conclusions

We conducted a meta-analysis of BM-derived cell therapy for patients with AMI and showed that this therapy was associated with long-term mortality benefits, although the effects of LV functional improvement and infarct size reduction were modest in short-term analyses. In this regard, to validate the therapeutic efficacy of cell therapy, future randomized clinical trials should include sufficient sample sizes with greater power to detect long-term clinical outcomes rather than surrogate short-term hemodynamic and image-based variables.

## Abbreviations

AMI: acute myocardial infarction
BM: bone marrow
CABG: coronary artery bypass grafting
G-CSF: granulocyte colony-stimulating factor
IHD: ischemic heart disease
LVEF: left ventricular ejection fraction
MNC: mononuclear cell
MRI: magnetic resonance imaging
MSC: mesenchymal stromal cell
PB: peripheral blood
PCI: percutaneous coronary intervention
RCT: randomized clinical trial
